# The Potential Anti-remodeling Effect of Paroxetine After Myocardial Infarction May Be Blunted by Beta-Blockers

**DOI:** 10.3389/fcvm.2022.887248

**Published:** 2022-07-11

**Authors:** Oriol Iborra-Egea, Alberto Aimo, Nicola Martini, Carolina Galvez-Monton, Silvia Burchielli, Giorgia Panichella, Claudio Passino, Michele Emdin, Antoni Bayes-Genis

**Affiliations:** ^1^ICREC (Heart Failure and Cardiac Regeneration) Research Programme, Health Sciences Research Institute Germans Trias i Pujol (IGTP), Barcelona, Spain; ^2^Hospital Universitari Germans Trias i Pujol, Badalona, Spain; ^3^Institute of Life Sciences, Scuola Superiore Sant’Anna, Pisa, Italy; ^4^Cardiology Division, Fondazione Toscana Gabriele Monasterio, Pisa, Italy; ^5^CIBER Cardiovascular, Instituto de Salud Carlos III, Madrid, Spain

**Keywords:** cardiac remodeling, myocardial infarction, paroxetine, artificial intelligence (AI), beta-blockers (BB)

## Abstract

**Background:**

Left ventricular (LV) remodeling consists in maladaptive changes in cardiac geometry and function following an insult such as ST-segment elevation myocardial infarction (STEMI). Interventions able to prevent LV remodeling after a STEMI are expected to improve the outcome of this condition. Paroxetine has inhibitory effects on GRK2, also known as beta-adrenergic receptor kinase 1 (ADRBK1). This drug does not yield beneficial effects on LV remodeling in patients with STEMI and LV ejection fraction ≤ 45%.

**Methods:**

We compared the molecular effects of paroxetine and drugs for neurohormonal antagonism (beta-blockers, angiotensin converting enzyme inhibitors/angiotensin receptor blockers, mineralocorticoid receptor antagonists), using a bioinformatic approach integrating transcriptomic data in a swine model of post-MI and available evidence from the literature and massive public databases.

**Results:**

Among standard therapies for MI, beta-blockers are the only ones acting directly upon GKR2, but the mechanism of action overlaps with angiotensin-converting enzyme inhibitors/angiotensin receptor blockers with respect to the AT2R-mediated anti-hypertensive response. Moreover, beta-blockers could have anti-fibrotic and anti-inflammatory effects through the regulation of myocyte-specific enhancer factors, endothelins and chemokines.

**Conclusion:**

The additive benefit of paroxetine on the background of the standard therapy for STEMI, which includes beta-blockers, is expected to be limited. Nonetheless, paroxetine becomes particularly interesting when a beta-blocker is contraindicated (for example, in hypotensive individuals) or poorly tolerated.

## Introduction

In-hospital and 30-day mortality associated with ST-segment elevation myocardial infarction (STEMI) has steadily declined over the last decades given the implementation of percutaneous coronary intervention and medical therapy for neurohormonal antagonism (1). This has been accompanied by a striking raise of patients who survive the acute event, but have an increased risk of heart failure (HF) and sudden chronic death (1). The rate of HF hospitalization rate is 4.4% in the first year after STEMI, and around 1.0% per year thereafter (2). In turn, patients who are hospitalized for HF during the first year have a risk of dying or being re-hospitalized for HF increased by 2-and 6-fold, respectively (2).

Left ventricular (LV) remodeling consists in maladaptive changes in cardiac geometry and function developing as a result to the loss of viable myocardium and an increased wall stress. This process involves multiple mechanisms such as tissue resorption, extracellular matrix (ECM) degradation, deposition of granulation tissue and vessel formation, followed by scar maturation, fibrosis and hypertrophy in the remote myocardium. LV remodeling eventually leads to impaired ventricular function and HF (3). Interventions able to prevent LV remodeling after STEMI are expected to improve the outcome of this condition.

A recent study found that paroxetine has no beneficial effects on LV remodeling in patients with STEMI and LV ejection fraction ≤ 45%. Specifically, paroxetine treatment was not associated with a recovery in LV ejection fraction compared to placebo (4). The rationale behind this trial was that paroxetine relieves LV remodeling when administered as a stand-alone medication to mice with MI (5). This effect was explained by an inhibitory effect of paroxetine on GRK2, also known as beta-adrenergic receptor kinase 1 (ADRBK1). GRK2 levels and activity have been reported to be enhanced in patients or in preclinical models of several disorders, including cardiac hypertrophy and HF, and to contribute to disease progression by a variety of mechanisms related to its multifunctional roles (6).

In this study, we aimed to elucidate whether the beneficial effects of pirfenidone observed in pre-clinical models do not translate into a clinical benefit because of overlapping mechanisms of action with other therapeutic approaches. Thus, we analyzed the molecular effects of drugs for neurohormonal antagonism (beta-blockers, angiotensin converting enzyme inhibitors/angiotensin receptor blockers, and mineralocorticoid receptor antagonists), which are recommended for patients with MI and systolic dysfunction.

## Methods

### Proteins Involved in Post-MI Remodeling or Modulated by Pirfenidone

Articles published over the last 10 years on the molecular pathophysiology of post-MI remodeling were searched in PubMed on October 7, 2020 with the following keywords: post(title) and [infarct*(title) or stroke (title)] and [myocardial (title) or cardiovascular (title) or cardiac (title)] AND [pathophysiology (Title/Abstract) OR pathogenesis (Title/Abstract) OR molecular (Title/Abstract)]. If the involvement of a protein candidate in post-MI remodeling was not well established, an additional PubMed search was performed, including all protein names according to UniProtKB.

### Post-MI Gene Expression Data in Swine

The Gene Expression Omnibus (7) and Array Express (8) public repositories were searched for gene expression data on post-MI remodeling with the following query (on December 9, 2020). No gene expression datasets from human myocardial tissue biopsies could be found. Our group previously employed microarray gene expression profiling of porcine cDNA to compare myocardial gene expression at baseline, 1, 4, and 6 weeks after surgically induced MI and in sham-operated controls (9). The swine transcriptomics were translated to their human equivalents *via* Reciprocal Best Hits (RBH) with BLAST and Gene Name Correspondence and the InParanoid database (10). Microarray data was processed using the GEO2R tool (7), and processed using the neqc method (11) and Linear Models for Microarray Analysis (12). We only considered genes with an adjusted *p*-value of < 0.01 (Benjamini-Hochberg false discovery rate), and log(fold-change) > 0.25. For introduction into the protein network, gene information was mapped one-to-one with proteins. In the end, 4,737 proteins were included. This information was used as experimental reference to model the algorithms and contrast the *in silico* findings to ensure their adhere to experimental evidence.

### Molecular Mechanisms of Action of Drugs

Artificial neural network (ANN) strategy can identify relationships among network regions (generalization) (13), allowing to infer the likelihood of a relationship between ≥ 2 sets of proteins. In this case, we tested each protein against the post-MI remodeling signature. Next, the model is validated using different data sets from the literature and databases. This system attempts to find the shortest distance between the 2 protein sets, generating a list of proteins ranked by their association with disease pathophysiology. ANN scores were calculated for angiotensin converting enzyme inhibitors (ACEi; target gene ACE, Uniprot P12821), angiotensin receptor blockers (ARB; target gene AGTR1, Uniprot P30556), beta-blockers (target gene ADRB1, Uniprot P08588), and mineralocorticoid receptor antagonists (MRA; target gene NR3C2, Uniprot P08235).

We then constructed a protein-protein interaction (PPI) network that contains all post-MI effectors to investigate the biological interactions of ACEi, ARB, beta-blockers, and MRA targets using the String platform (14). All scores rank from 0 to 1, with 1 being the highest possible confidence in judging the interaction as true. Here we used a minimum score of 0.9 to investigate how ACEi/ARB, beta-blockers, and MRA targets interact with our molecular characterization of post-MI remodeling, which includes 222 proteins. Unsupervised clustering was performed using a K-Means approach, with the number of clusters (*K* = 10) determined through the elbow method.

## Results

### Beta-Blockers Selectively Target GRK2 Signaling Pathway

**FIGURE 1:**
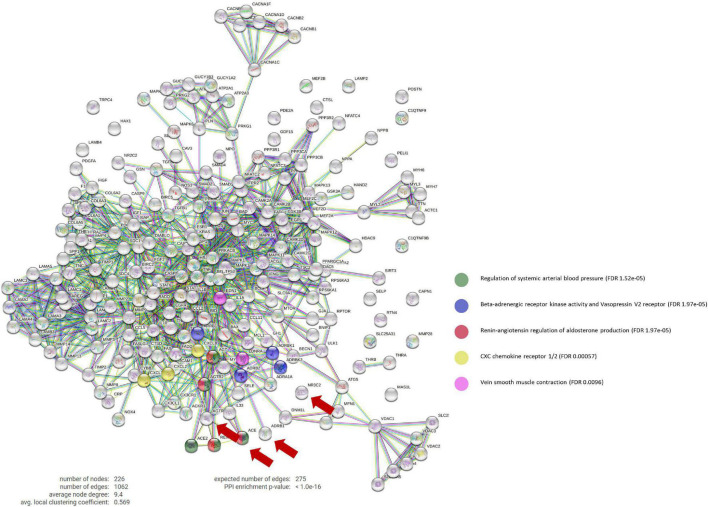
Protein-Protein interaction network. PPi network that contains the interactions between the main targets of ACEi, BB, MRA, and ARB treatments (ACE1, ADRB1, NR3C2, and AGTR1, respectively) and our curated molecular characterization of post-MI remodeling (which includes 222 proteins). BB main effect involve the Beta-adrenergic receptor kinase activity and vasopressin V2 receptor biological pathways (in blue), which include GPRK2 modulation. Number of nodes: 226; Number of connections: 1,062; Expected number of random connections: 275; PPi Enrichment *p*-value: < 1.0e-16; PPi, Protein-protein interaction; ACEi, angiotensin converting enzyme inhibitors; BB, beta-blockers; MRA, mineralocorticoid receptor antagonists; ARB, angiotensin receptor blockers.

The constructed ANN network identified GRK2 signaling pathway as one of the main targeted mechanisms by gold-standard drugs during the pathology characterization. Through a PPI analysis, we found that beta-blockers strongly modulate GRK2 activity (blue dots in Figure 1), as well as venous smooth muscle contraction, which in turn is related to the renin-angiotensin-aldosterone (RAA) pathway, and includes several family-members of GRK2.

Moreover, this targeted mechanism of action seems to be specific of beta-blockers, as only beta-blockers appear to act directly upon GRK2.

### ACEi Reinforce Beta-Blockers Mechanism of Action Upon GRK2 by AT2R-Mediated Vasodilation

By inhibiting AGTR1, ACEi disrupt the ACE/Angiotensin II/AT1R molecular cascade and allows AT2R to exert vasodilation functions. GRK2 is described to desensitize beta-adrenergic receptors to G-protein coupled receptors (GPCRs), such as AT2R. Here we identified that this binding blocks G-protein coupling and leads to internalization of AT2R *via* the b-arrestin Arrb1. AT2R then cannot compete with AT1R, which results in vasoconstriction and thus increases arterial blood pressure.

By decreasing the levels of Angiotensin II/AT1R complex, ACEi balances AT2R expression, induce vasodilation through a nitric oxide/bradykinin-dependent pathway and exert a synergistic effect upon GRK2 direct inhibition by beta-blockers.

ARBs work very similarly to ACEi, but act by blocking AT1R directly (which also increases AT2R effect), inciting vasodilation, but do not compete directly with the same proteins as beta-blockers.

MRAs have not been found to act upon any common signaling pathway with GRK2 or beta-blockers.

### Regulation of Myocyte-Specific Enhancers, Endothelins and Chemokines Drive Anti-fibrotic and Anti-inflammatory Properties

Our analysis also shows that beta-blockers could also have anti-fibrotic and anti-inflammatory effects in post-MI LV remodeling with myocyte-specific enhancer factors (MEF2A, MEF2C, MEF2D), endothelins (EDN and EDNRA) and a series of chemokines, such as CXCL1, CXCL2, and IL-8, as mediators.

Upon the MI insult, MEFs transmits a stress response in cell growth, survival and apoptosis *via* CAMKII and the MAPK pathways. At the same time, our analysis uncovers the important role of RELA (P65) as a mediator of this pro-inflammatory cascade by directly regulating endothelial cells and T-cells expression of EDN and EDNRA or CXCL1 and CXCL2, respectively.

## Discussion

In this manuscript, we wanted to investigate why paroxetine could have failed to improve LV remodeling in STEMI patients with LVEF < 45% in a recent trial (4), despite the very interesting pre-clinical findings studies (5), attributed to the inhibition of GRK2.

We hypothesized that this beneficial effect seen in mice could be lost when translated to the clinical setting because STEMI patients already receive other pharmacological treatments that could be masking this effect. For this reason, we wanted to investigate if these other treatments could already be acting upon GRK2 or similar processes.

Among standard therapies for MI, beta blockers are the only ones acting directly upon GKR2, but the mechanism of action overlaps with angiotensin-converting enzyme inhibitors/angiotensin receptor blockers with respect to the AT2R-mediated anti-hypertensive response.

AT2R is a G-protein-coupled receptor and the physiological function of GPCRs is regulated by G protein–coupled receptor kinases (GRKs). The blockade of beta-adrenergic receptors significantly increases the affinity of GPCR for b-arrestin in the cytoplasm, leading to the formation of a complex and thereby terminating signal transduction (15).

We were able to incorporate hundreds of thousands of protein interactions into our models, making them the most robust possible and the most trustworthy considering the current state of scientific knowledge. We report that the inhibitory effects of paroxetine and beta-blockers are overlapping, and the additive benefit of paroxetine on the background of the standard therapy for STEMI, which includes beta-blockers (1), is expected to be limited. This conclusion is in agreement with the proposed limited efficacy of paroxetine modulation in the post-MI setting. Then, therapy with paroxetine may become particularly interesting when a beta-blocker is contraindicated (for example, in hypotensive individuals) or when is poorly tolerated.

Future studies should investigate the possibility to replace beta-blockade with selective modulation of GRK2 to reverse LV remodeling after a STEMI.

## Limitations

Our approach has some limitations. The AI-based models used in this study only incorporate information that has already been described and demonstrated experimentally. As knowledge on paroxetine and post-MI omics information is rapidly evolving, it is likely that the mechanism of action is more multifactorial than currently presented. Any future discovery of protein function or new information that changes what we know about the disease or the drugs under study cannot be captured and may change the results presented here. Finally, the pharmacological effect of these drugs upon STEMI could encompass, and be affected by, other molecular signaling cascades, as well as by a wide variety of physiological affected pathways. Although our models show that GRK2 signaling is the most robust pathway affected, it is likely that other pathways may be involved as well.

## Data Availability Statement

The datasets presented in this study can be found in online repositories. The names of the repository/repositories and accession number(s) can be found below: https://www.ncbi.nlm.nih.gov/geo/, GSE34569.

## Ethics Statement

The animal study was reviewed and approved by the Animal Experimentation Unit Ethical Committee of the Catalan Institute of Cardiovascular Sciences (ICCC) [Permit No. 4563; Departament de Medi ambient i Habitatge (DMAH), Generalitat de Catalunya]. The protocol complied with all guidelines concerning the use of animals in research and teaching, as defined by The Guide for the Care and Use of Laboratory Animals (NIH Publication No. 80–23, revised 1996).

## Author Contributions

OI-E and AA contributed to conception and design of the study. OI-E performed all bioinformatic analyses. AA wrote the first draft of the manuscript. OI-E, AA, AB-G, and ME wrote sections of the manuscript. AB-G and ME supervised the study. All authors contributed to manuscript revision, read, and approved the submitted version.

## Conflict of Interest

The authors declare that the research was conducted in the absence of any commercial or financial relationships that could be construed as a potential conflict of interest.

## Publisher’s Note

All claims expressed in this article are solely those of the authors and do not necessarily represent those of their affiliated organizations, or those of the publisher, the editors and the reviewers. Any product that may be evaluated in this article, or claim that may be made by its manufacturer, is not guaranteed or endorsed by the publisher.
